# QTL Mapping of Fiber- and Seed-Related Traits in Chromosome Segment Substitution Lines Derived from *Gossypium hirsutum* × *Gossypium darwinii*

**DOI:** 10.3390/ijms25179639

**Published:** 2024-09-05

**Authors:** Wenwen Wang, Yan Li, Mingmei Le, Lixia Tian, Xujing Sun, Rui Liu, Xin Guo, Yan Wu, Yibing Li, Jiaoyun Zhao, Dajun Liu, Zhengsheng Zhang

**Affiliations:** 1 Engineering Research Center of South Upland Agriculture, Ministry of Education, Southwest University, Chongqing 400715, China; www20216024@swu.edu.cn (W.W.); liyan88878@wchscu.cn (Y.L.); zwmnm1871@163.com (M.L.); 13022388566@163.com (L.T.); 18337576882@163.com (X.S.); liurui.4080@163.com (R.L.); 15890509142@163.com (X.G.); wy1516847935@163.com (Y.W.); liyibing2023@163.com (Y.L.); 18435609252@163.com (J.Z.); liudajun_ren_ren@163.com (D.L.); 2 Chongqing Key Laboratory of Crop Molecular Improvement, Southwest University, Chongqing 400715, China

**Keywords:** *G. hirsutum*, *G. darwini*, chromosome segment substitution lines, fiber- and seed-related traits, QTL

## Abstract

A narrow genetic basis limits further the improvement of modern *Gossypium hirsutum* cultivar. The abundant genetic diversity of wild species provides available resources to solve this dilemma. In the present study, a chromosome segment substitution line (CSSL) population including 553 individuals was established using *G. darwinii* accession 5-7 as the donor parent and *G. hirsutum* cultivar CCRI35 as the recipient parent. After constructing a high-density genetic map with the BC_1_ population, the genotype and phenotype of the CSSL population were investigated. A total of 235 QTLs, including 104 QTLs for fiber-related traits and 132 QTLs for seed-related traits, were identified from four environments. Among these QTLs, twenty-seven QTLs were identified in two or more environments, and twenty-five QTL clusters consisted of 114 QTLs. Moreover, we identified three candidate genes for three stable QTLs, including *GH_A01G1096* (*ARF5*) and *GH_A10G0141* (*PDF2*) for lint percentage, and *GH_D01G0047* (*KCS4*) for seed index or oil content. These results pave way for understanding the molecular regulatory mechanism of fiber and seed development and would provide valuable information for marker-assisted genetic improvement in cotton.

## 1. Introduction

Cotton, one of the most widely cultivated cash crops in the world, mainly provides natural fiber for modern textile industries. Furthermore, it is also a significant source of edible oil, feed, and biofuel [1,2]. The wide range of uses of cotton have linked it closely with people’s daily lives. To meet the conditions of the reduction of arable land, the rapid development of the textile industry, and the demands of better clothing quality, it is becoming increasingly relevant to cultivate and popularize cotton varieties with superior performance, including their high yield and the high quality of their fiber and seed [3]. However, fiber- and seed-related traits are quantitative traits controlled by multiple genes and are simultaneously subjected to environmental factors, resulting in the slow progress of genetic improvement [4].

The *Gossypium* genus consists of 52 species, including 45 diploids and 7 allotetraploids [5]. *G. hirsutum*, a widely cultivated cotton species, produced 95% cotton in the world [6]. However, the narrow genetic basis of modern upland cotton cultivars has resulted from long-term domestication and artificial selection has limited the development of cotton research and breeding. It has become a continuous pursuit to explore new germplasm resources and discover novel alleles relevant to important agronomic traits in cotton research [7]. Wild cotton species have gained excellent characteristics and adaptation mechanisms to resist various adverse factors under long-term natural selection, which are potential resources for broadening the genetic diversity of *G. hirsutum* [5]. There are five wild cotton species, including *G. tomentosum*, *G. mustelinum*, *G. darwinii*, *G. ekmanianumsi*, and *G. stephensii* [8]. To date, three wild cotton species (*G. mustelinum*, *G. darwinii*, and *G. tomentosum*) have been used for genetic breeding and improvement [7,9,10]. *G. darwinii*, one of the allotetraploid wild cotton species distributed on the Galapagos Islands [11], is closely related to *G. barbadense* but quite different from the cultivated *G. barbadense* and *G. hirsutum*. It has the advantage of fine fiber, drought resistance, verticillium wilt resistance, and so forth [12]. It is of great importance to explore favorable alleles from *G. darwinii* and be applied for upland cotton cultivar improvement.

Due to the barrier of interspecific incompatibility, segregation distortion, suppression of recombination, and linkage drag need to be overcome first [13,14,15]; it is quite difficult to transfer these favorable traits directly into cultivated cotton by conventional breeding. Chromosome segment substitution lines (CSSLs) are optimal tools for transferring favorable genes into cultivated cotton. After a series of backcrossing and self-crossing events, these CSSLs are distinct from each other by several DNA polymorphisms, which were substituted by different segments from the donor parent. Except for the comparatively small, substituted segments, all the other fragments of the chromosomes are derived from the receptor parent. Construction of chromosome segment substitution lines will provide the foundation for the genetic dissection of important traits and facilitate utilization in breeding by marker-assisted selection (MAS) [16]. In the previous study, an introgression line (IL) population including 105 lines was obtained by crossing *G. darwinii* Watt with Ejing1, and 40 QTLs controlling fiber quality were identified [7]. However, a small number of markers and lower introgression percentages limited the application of this introgression line (IL) population in further research or cotton breeding.

In this study, a high-density genetic linkage map was first constructed based on a BC_1_ population developed from the CCRI 35 and *G. darwinii* accession 5-7. A set of 553 progeny CSSLs of advanced generations were obtained. Then, we genotyped these CSSLs and analyzed the distribution of chromosome fragments introgressed from *G. darwinii*. QTL mapping of cotton seed- and fiber-related traits were detected based on phenotypic values in multi-environments. Genes in the confidence interval were analyzed to predict the candidate genes of stable QTLs or QTL clusters.

## 2. Results

### 2.1. Genetic Map Construction

**Figure 1:**
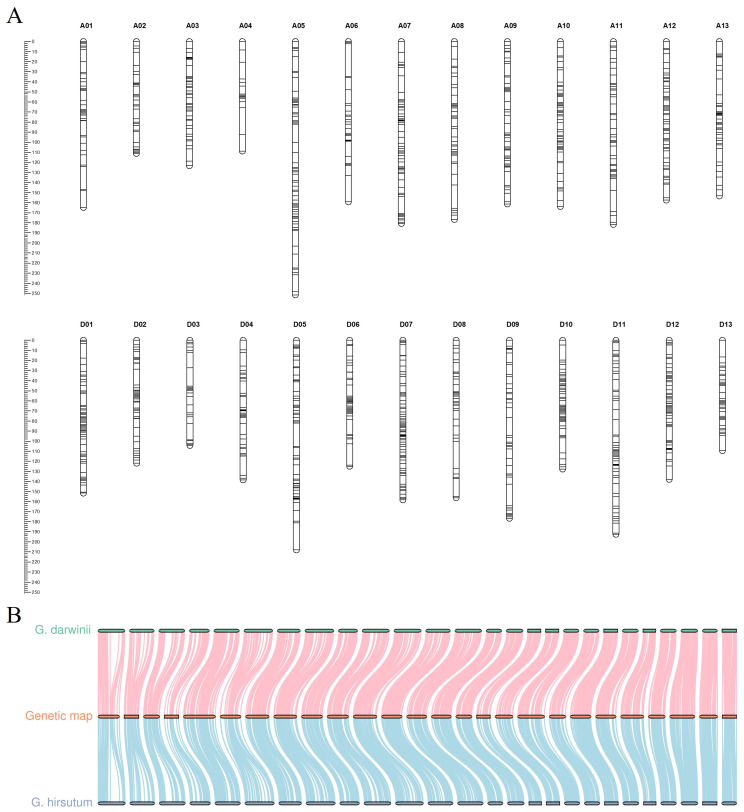
The interspecific genetic map of the (CRRI35 × *G. darwinii* 5-7) BC_1_ population. (**A**) The genetic map of (CRRI35 × *G. darwinii*) BC_1_ population, (**B**) collinearity analysis between the genetic map and two associated physical maps of *G. hirsutum* and *G. darwinii*.

A total of 6009 primers were first screened for the polymorphism between CCRI35 and *G. darwinii* 5-7. Subsequently, 2178 primer pairs with clear polymorphism were carried to genotype the BC_1_ population of (CCRI35 × *G. darwinii* 5-7) × CCRI35. Finally, a high-density genetic map including 2309 loci was constructed. This map covered 4002.16 cM with an average distance of 1.73 cM between adjacent markers. The A_t_ subgenome contained 1006 loci spanning 2094.06 cM and the D_t_ subgenome contained 1303 loci spanning 1908.10 cM. The genetic distance of 26 chromosomes were ranged from 104.30 cM (ChrD03) to 251.60 cM (ChrA05) (Figure 1A; Table S1). Moreover, the genetic map showed high collinearity with both the *G. hirsutum* and *G. darwinii* reference genomes (Figure 1B).

### 2.2. Introgressive Segments Analysis of the CSSLs

**Figure 2:**
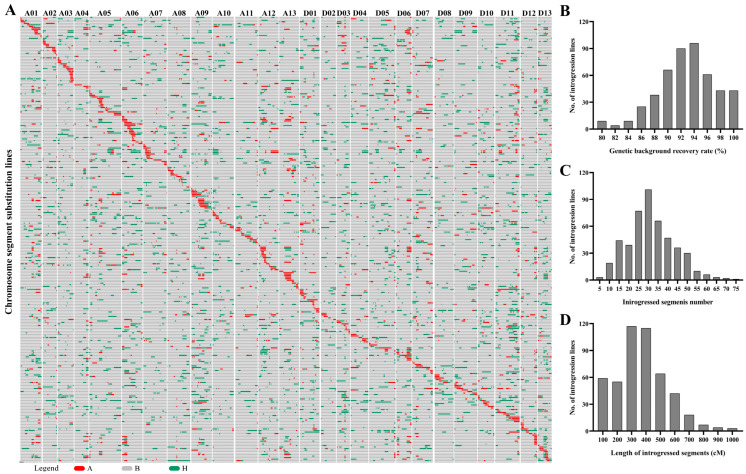
Genetic constitution and introgressive segments of the CSSLs. (**A**) Distribution of introgressed segments in the CSSLs on the 26 chromosomes. A and H represent homozygous and heterozygous chromosome segments from the donor parent *G. darwinii* 5-7, respectively; (**B**) represents homozygous chromosome segments from the recurrent parent CCRI35. (**B**–**D**) Genetic background recovery rate and the number and length of the introgressed segments in the CSSLs population.

A chromosome segment substitution line (CSSL) population including 553 individuals was constructed in this study. Then, a total of 551 markers evenly distributed on 26 chromosomes were selected to test the genotype of 553 CSSL lines. The coverage of the introgressed segments in the genome and percentage of genome coverage were investigated. As shown in Figure 2A, the introgressed segments covered 26 whole chromosomes. The number of introgressed segments in each line ranged from 2 to 73 (Figure 2C). The genetic distance of the introgressed chromosome segments from *G. darwinii* for each CSSL ranged from 15.77 to 914.47 cM, with an average of 321.13 cM (Figure 2D). The coverage of the introgressed segments ranged from 0.4% to 23.2%, with an average of 10.27%.

### 2.3. Characterization of Phenotypic Performance

**Figure 3:**
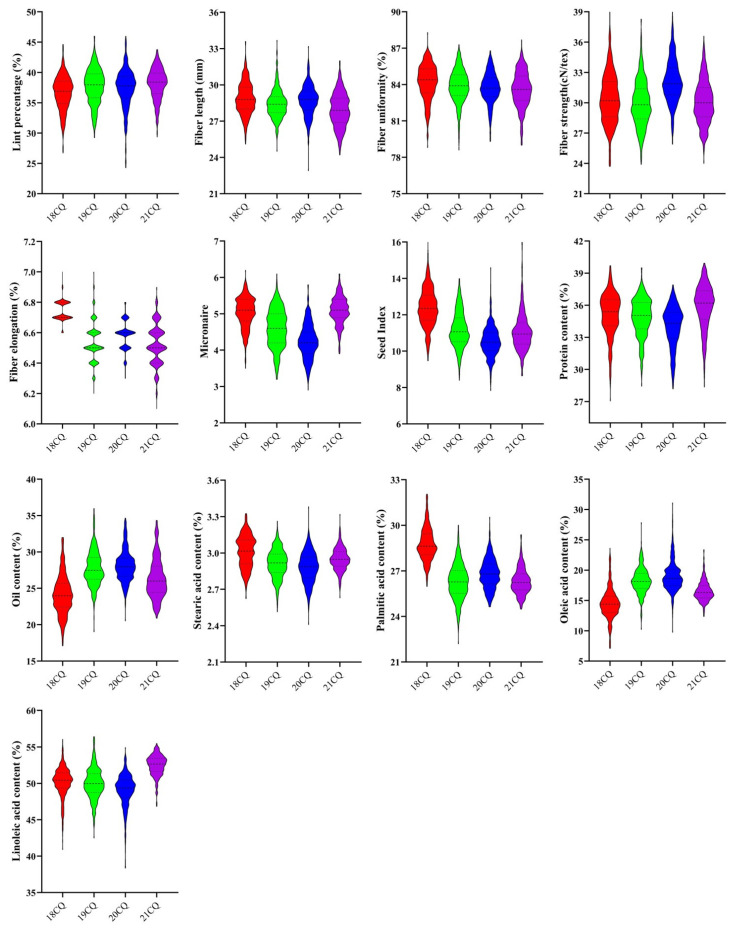
The phenotypic performance of CSSLs across four environments. 18CQ, 19CQ, 20CQ, and 21CQ represent 2018, 2019, 2020, and 2021 in Chongqing.

Due to the specific growth habit of *G. darwinii*, only the recurrent parent CCRI35 together with CSSLs population were planted in four environments. Descriptive statistics of phenotypic traits of the CSSLs population are presented in Table S1. Among the CSSLs population, all traits were distributed continuously with some fluctuations in different environments (Figure 3). The absolute skewness of all traits in the four environments was less than one, thus following a normal distribution. Moreover, the results of the ANOVA test showed that both environmental and genotypic effects have a significant impact on all examined traits (Table S2).

**Figure 4:**
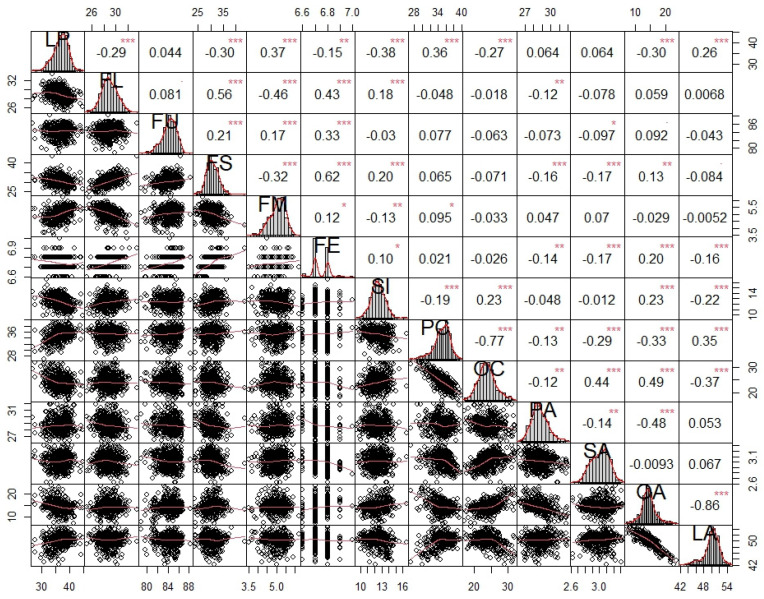
Correlation analysis among fiber- and seed-related traits. The values in the correlation matrix represent Pearson’s correlation coefficient. Positive values represent positive correlations and negative values represent negative correlations. *, **, and ***, significant correlation at the 0.05, 0.01, and 0.001 probability levels, respectively. LP, lint percentage (%); SI, seed index (g); FL, fiber length (mm); FS, fiber strength (cN/tex); FU, fiber uniformity (%); FM, fiber micronaire; FE, fiber elongation (%); PC, protein content (%); OC, oil content (%); PA, palmitic acid content (%); SA, stearic acid content (%); OA, oleic acid content (%); LA, linoleic acid content (%).

Correlation analysis of all traits was conducted and is visualized in Figure 4. Among the six fiber-related traits, LP and FM show a strong negative correlation with FL and FS. A significantly positive correlation occurred among FL, FS, and FE. Among the seven seed-related traits, SI was significantly positively correlated with OC and OA but negatively correlated with PC and LA. A strong negative correlation existed between OC and PC, OA, and PA, as well as OA and LA. Moreover, highly significant correlations were observed between fiber-related traits and seed-related traits. Six paired traits (LP and PC/LA, FL and SI, FS and SI/OA, and FE and OA) showed significant positive correlations. Four paired traits (LP and SI/OC/OA, FL and PA, FS and PA/SA, and FE and PA/SA/LA) showed significant negative correlations.

### 2.4. QTL Mapping for Fiber- and Seed-Related Traits

**Figure 5:**
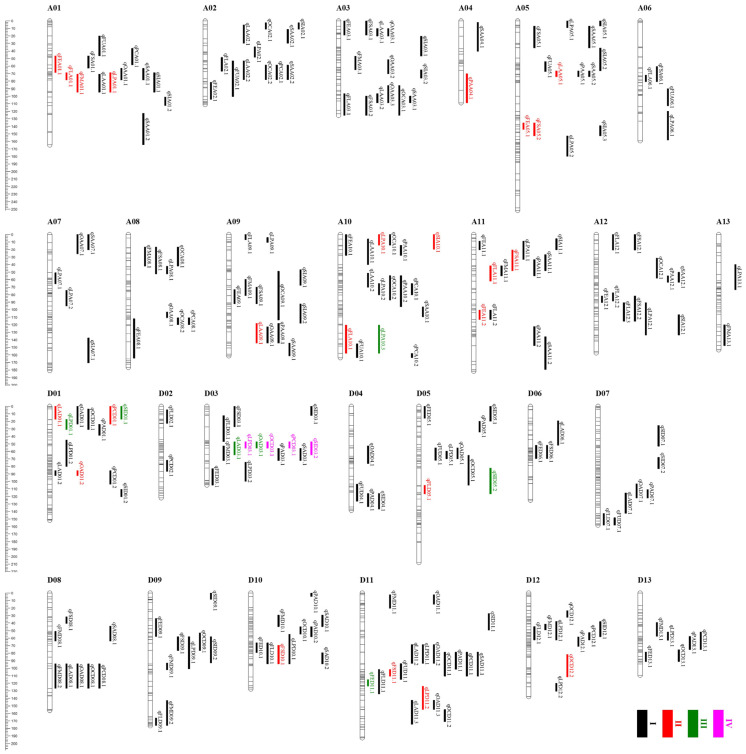
QTLs detected on all chromosomes. I, II, III, and IV indicate that QTL were detected in one environment, two environments, three environments, and four environments, respectively.

A total of 104 QTLs, including 27 QTLs for lint percentage and 77 QTLs for five fiber quality traits, were identified with the phenotypes from four environments (Figure 5; Table S3). Of the QTLs, 19 QTLs were detected in two or more environments and considered as stable QTLs. Three QTLs (*qLP_A10.3_*, *qLP_D01.1_*, and *qFE_D11.1_*) were identified across three environments, and *qLP_D03.1_* was detected across four environments. The favorable alleles of all stable lint percentage QTLs were contributed by CCRI35, whereas the favorable alleles of fiber quality related QTLs were derived from *G. darwinii* 5-7.

Moreover, we detected 131 QTLs controlling seed-related traits, including 27 for SI, 20 for OC, 13 for PC, 17 for OA, 17 for LA, 18 for PA, and 19 for SA (Figure 5; Table S3). Of the QTLs, eight QTLs were detected across two environments. Four (*qSI_D01.1_*, *qSI_D05.2_*, *qOA_D03.1_*, *qLA_D03.1_*) and three (*qOC_D03.1_*, *qPC_D03.1_*, *qSI_D03.2_*) QTLs were found across three and four environments, respectively. The stable QTLs controlling SI, OA, and PA had positive additive effects derived from *G. darwinii* 5-7, and the stable QTLs controlling PC and LA deriving from CCRI35.

Based on the position of 235 fiber- and seed-related QTLs, we found 25 QTL clusters be made up of 114 QTLs (Figure 5; Table 1). These clusters were distributed on 17 chromosomes, including 9 clusters on the At subgenome and 8 clusters on the Dt subgenome. Eleven out of twenty-five clusters contained five or more QTLs. Both A01-cluster-2 and D11-cluster-1 included seven QTLs. D03-cluster-1 contained nine QTLs, and six out of nine QTLs were found in three or more environments. Eleven of the QTL clusters contained at least one stable QTL, and five QTL clusters contained more than two stable QTLs. Four QTL clusters (A02-cluster-1, A09-cluster-1, A12-cluster-1, and D01-cluster-2) contained only seed-related QTLs, while others were associated with both fiber- and seed-related traits. These QTL clusters combined with stable QTLs above would provide potential loci for further functional research and cotton breeding.

### 2.5. Functional Annotation of Candidate Genes in QTL Clusters

**Figure 6:**
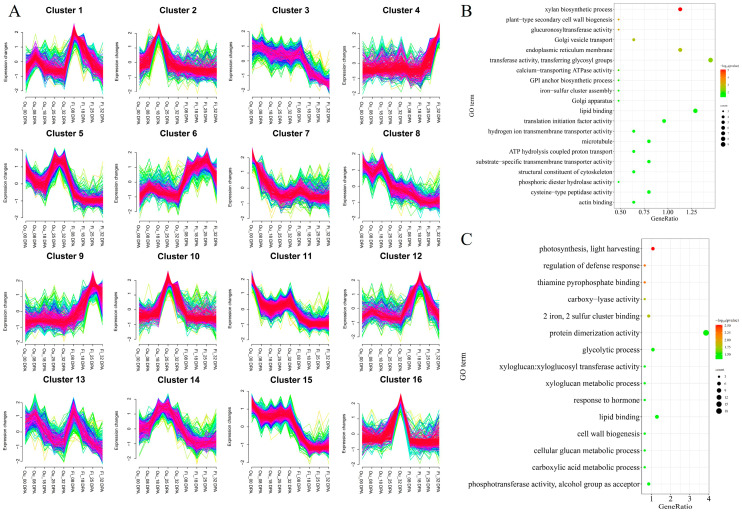
Expression patterns of candidate genes in 16 QTL clusters in CCRI35. (**A**) Expression profile of candidate genes. The color represents the density of gene in this cluster. Green and red represent low and high density, respectively. (**B**) GO annotation of candidate genes associated with fiber initiation. (**C**) GO annotation of candidate genes associated with seed development and oil accumulation.

According to the annotation of the reference genome [17], the confidence intervals of 25 QTL clusters contained 10,747 genes. Among these genes, 6172 genes were expressed in the fiber or ovule development of CCRI35 (Table S4). Based on the expression profiles at fiber and ovule development stages, 6172 genes were classed into 16 clusters (Figure 6A). The genes in the 16 clusters show different expression patterns at different development stages.

In Cluster 7, Cluster 8, Cluster 11, and Cluster 15, the genes showing high expression at 0 days post-anthesis (DPA) may be associated with fiber initiation and finally lint percentage (Figure 6A). Gene Ontology enrichment analysis showed 1357 genes were enriched in 61 terms, of which 4 genes were annotated in Rho guanyl-nucleotide exchange factor activity (GO:0005089), 12 genes in kinesin complex (GO:0005871), microtubule-based process (GO:0007018 and GO:0003777) (Figure 6B; Table S5). The genes in Cluster 1, Cluster 6, and Cluster 13 were highly expressed at 8 DPA of fiber, which was related to fiber elongation or fiber length. Small GTPase-mediated signal pathway (GO:0007264, GO:0003924, GO:0008536), calcium-dependent phospholipid binding (GO:0005544), and potassium ion transmembrane transporter activity (GO:0015079) were significantly enriched in these clusters (Figure S1; Table S6). The genes in Cluster 4, Cluster 9, and Cluster 12 were mainly expressed at the 18DPA, 25DPA, and 32DPA of fibers (Figure 6A). The GO terms relating secondary cell wall deposition, including xylan biosynthetic process (GO:0045492), plant-type secondary cell wall biogenesis (GO:0009834), and glucuronosyltransferase activity (GO:0015020), were significant enriched (Figure S2; Table S7).

It is reported that seed oil accumulation mainly occurs at the late stage of ovule development [18]. In Cluster 2, Cluster 8, and Cluster 14, genes expressed at 8 DPA and 18DPA of ovule were mainly related to seed size (Figure 6A). Cell cycle arrest (GO:0007050) and glycogen biosynthetic process (GO:0005978) were significantly enriched (Figure S3; Table S8). While genes in Cluster 10 and Cluster 16, showing high expression at the late stage of ovule development, may be associated with seed size and oil accumulation (Figure 6A). The GO terms related to seed development and oil accumulation were significantly enriched, such as the xyloglucan metabolic process (GO:0010411), cellular glucan metabolic process (GO:0006073), xyloglucosyl transferase activity (GO:0016762), and lipid binding (GO:0008289) (Figure 6C; Table S9).

## 3. Discussion

### 3.1. Utilization of G. darwinii and Its Related CSSLs

Compared with *G. barbadense* and *G. tomentosum*, the research on *G. darwinii* is relatively backward. *G. darwinii* possessed a variety of excellent characteristics to adapt to the wicked environment, such as fine fiber, drought resistance, verticillium wilt resistance, and so forth. In the present study, chromosome segments spanning almost the entire *G*. *darwinii* genome were introgressed into *G. hirsutum* through multiple-generation backcross (Figure 2A). Based on this CSSL population, a great many QTLs controlling fiber- and seed-related traits were identified (Table S3). The QTLs associated with drought resistance and verticillium wilt resistance will be analyzed in future research. These QTLs and CSSLs provide a foundation for in-depth research on fiber and seed development. Moreover, some chromosome segment substitution lines possessing favorable allele for one or more target traits could be directly utilized as superior varieties.

### 3.2. Sources and Effects of Favorable Alleles

Introducing favorable alleles from other species into *G. hirsutum* cultivars is one of the major strategies in the breeding practice of cotton. Throughout this process, favorable allele identification is a crucial step. The number of favorable alleles had been reported in *G. barbadense*, *G. mustelium,* and *G. tomentosum* [9,10,19,20,21,22,23], which is a viable resource for improving fiber quality of *G. hirsutum*. In this study, a total of 235 QTLs associated with 13 fiber- and seed-related traits were identified. Among these QTLs, 102 favorable alleles of QTLs were from *G*. *darwinii* and 133 favorable alleles of QTLs were from CCRI35. Most of the favorable alleles for lint percentage were derived from *G. hirsutum* cultivar CCRI35 (22/27), which may be the result of a pursuit of fiber yield during the domestication of cotton. Meanwhile, most of the favorable alleles for fiber quality were from *G*. *darwinii* (60/77). It is noteworthy that among the detected stable QTLs (*qFL_A01.1_*, *qFL_A10.1_*, *qFL_A11.1_*, *qFL_D05.1_*, *qFS_A05.2_*, *qFS_A11.1_*, *qFS_D10.1_*, *qFS_D11.1_*, *qFM_A01.1_*, *qFE_A01.1_*, *qFE_A05.1_*, *qFE_A11.2_*, and *qFE_D11.1_*) related to fiber quality, the favorable alleles were all derived from *G*. *darwinii* (Table S3). Moreover, *G*. *darwinii* also provided the favorable alleles of 75 seed-related QTLs, including stable QTL (*qSI_A10.1_*, *qSI_D01.1_*, *qSI_D03.2_*, *qSI_D05.2_*, and *qOC_D03.1_*) associated with seed index and oil content. The abundant favorable alleles of *G*. *darwinii* can help to improve fiber quality, seed size, and oil content and are of great significant research value for the improvement of upland cotton varieties.

### 3.3. Comparison of CSSLs between G. darwinii and G. barbadense

In comparison with other tetraploid cotton, *G*. *darwinii* and *G*. *barbadense* show the most recent divergence (~0.20 Ma) and are considered to be descendants from a common ancestor [11,24]. There is a close kinship and some divergence between *G*. *darwinii* and *G*. *barbadense*. In our previous study, a chromosome segment substitution line population was established using *G. barbadense* cultivar Pima S-7 as the donor parent and *G. hirsutum* cultivar CCRI35 as the recipient parent [23]. It shares common recurrent parent (*G. hirsutum* cultivar CCRI35) with the population developed in this study. The introgressive segments of either *G. barbadense* or *G. darwinii* span almost the whole genome in their CSSL populations (Figure 2). Totals of 105 and 104 QTLs were identified based on the CSSLs population of *G. barbadense* or *G. darwinii*, respectively. Among these QTLs, 34 QTLs (~32%), including 9 QTLs for lint percentage and 25 QTLs for fiber quality, were detected in both two CSSL populations. Compared with *G. barbadense*, there are less common QTLs for fiber-related traits in *G. tomentosum* CSSL population [10]. These results may be the result of the closer genetic relationship between *G*. *darwinii* and *G*. *barbadense* compared to others. Meanwhile, the specific QTLs in each CSSLs population indicated the divergence of *G*. *darwinii* or *G*. *barbadense*. This divergence would provide novel favorable alleles for upland cotton improvement. Moreover, *qLP_D03.1_* was also detected in multiple populations with different male parent including wild and semi-wild species [6,10,23,25]. It indicated that the allele of *qLP_D03.1_* is an ideal allele for improving fiber yield and may be a key watershed in the domestication of upland cotton cultivar.

### 3.4. Identification of Candidate Genes Associated with Stable QTLs

According to the physical distance of confidence intervals, three stable QTLs with relatively small intervals were selected for candidate gene identification. *qFL_A01.1_*, a member of A01-cluster-2, was positioned at 13.79–34.21 Mb on A01. In this region, an auxin response factor (*ARF5*, GH_A01G1096) associated with auxin signal was located. There is an SNP in the coding region of *GoARF5* between the two parents. It was reported that *GhARF5* regulates the expression of *GhROP7*/*GhRAC13*, and then affects the onset of secondary growth [26]. In A10-cluster-1, two stable QTLs (*qLP_A10.1_* and *qSI_A10.1_*) were mapped to a 1.97 Mb interval on A10, where *PDF2* (GH_A10G0141), a homeobox-leucine zipper protein, was located. Three nonsynonymous mutations were found between *G. hirsutum* and *G*. *darwinii*. Cotton *PDF2* was highly expressed in ovule epidermis and fiber cells. Knockout *PDF2* significantly decreased fiber initials on 0 DPA ovules [27]. Therefore, *GoARF5* and *GoPDF2* might be the possible underlying gene of *qFL_A01.1_* and *qLP_A10.1_*, respectively, and play a key role in regulating fiber development.

*qOCD01.1*, detected in three environments and overlapping with *qLP_D01.1_* and *qSI_D01.1_* in D01-cluster-1, was anchored on 165366-1717322bp on D01. *KCS4* (GH_D01G0047), a gene encoding an enzyme involved in very-long-chain fatty-acid (VLCFA) synthesis, which is a branch point in the regulation of triacylglycerol synthesis [28]. A base deletion in the first exon results in frame shift and thus an extension of 137 amino acid in *G*. *darwinii* 5-7. Therefore, *KCS4* here might be the underlying gene of *qOC_D01.1_*, which participates in the oil accumulation in cotton seed development.

## 4. Materials and Methods

### 4.1. Plant Materials

The BC_1_ populations for genetic map construction were developed from the cross and backcross between donor parent *G. darwinii* accession 5-7 and recurrent parent CCRI35 in Chongqing, China, in 2013. *G. darwinii* accession 5-7 was provided by the Institute of Cotton Research of Chinese Academy of Agriculture Sciences. CCRI35 is a *G. hirsutum* cultivar with characteristics of high yield and disease resistance [29]. After further backcross with recurrent parent CCRI35 and selfing, the CSSL (BC_3_F_2_) population, including 553 individuals, was generated in Chongqing province, China, in 2017. Subsequently, the generations from BC_3_F_2:3_ to BC_3_F_2:6_ were planted from 2018 to 2021 in Chongqing province, China. The weather information for cotton cultivation from 2018 to 2021 is provided in Table S10.

### 4.2. Phenotypic Collection and Analysis

The naturally opened bolls of 553 CSSLs were hand-harvested from BC_3_F_2:3_ to BC_3_F_2:6_ in Chongqing. The fiber-related traits include lint percentage (LP, %), fiber length (FL, mm), fiber strength (FS, cN/tex), fiber uniformity (FU, %), fiber elongation (FE, %), and fiber micronaire (FM). The seed-related traits contain seed index (SI, g), kernel protein content (PC, %), protein oil content (OC, %), protein palmitic acid content (PA, %), protein stearic acid content (SA, %), protein oleic acid content (OA, %), and protein linoleic acid content (LA, %). The Lint percentage and seed index were manually weighted. Cottonseed nutrient quality traits (PC, OC, PA, SA, OA, and LA) were measured using the calibration model of near infrared reflectance spectrum (NIRS) established in our laboratory [10]. Fiber quality traits (FL, FS, FU, FM, and FE) were tested with High Volume Instrument (HVI) 900 instrument (Uster ^®^ Hvispectrum, Spinlab, Knoxville, TN, USA).

The statistical analysis and the analysis of variance (ANOVA) were performed in Microsoft Excel (Office 2016), and the R-4.3.0 software were carried out for correlation analysis and visualization.

### 4.3. Genetic Map Construction and Collinearity Analysis

The primers for genetic map construction were selected from a high-density interspecific genetic map between *G. hirsutum* and *G. barbadense* [30]. The primers were first screened for polymorphisms between CCRI35 and *G. darwinii* accession 5-7. Polymorphic markers were used to genotype the BC_1_ population and further construct the genetic linkage map using JoinMap 4.0 [31].

The physical map was constructed according to the physical positions of polymorphic markers. The physical positions of all markers were obtained by aligning the primer sequences to *G. hirsutum* and *G. darwinii* reference genomes using BLAST + 2.15.0 software [24]. Then, a python library JCVI was used to analyze and illustrate the relationship between genetic map and two physical maps [32].

### 4.4. Detection of Introgressive Chromosome Segments

Based on the genetic map constructed in this study, markers evenly distributed on the genetic map were selected to genotype the 553 individuals of CSSL population. The average interval between the two markers was approximately 10 centimorgans (cM). GGT2.0 software [33] was applied to analyze the characteristic of chromosomal introgressed segments (the background recovery rate of the CSSLs and the number and length of introgressed segments) with default parameters.

### 4.5. Identification of Fiber- and Seed-Related QTLs

MapQTL 6.0 was applied to map QTLs by Multiple-QTL model (MQM), with a threshold of LOD ≥ 2.0 [34]. Positive additive effects indicated that *G. darwinii* contribute to the favorable alleles of QTLs, whereas negative additive effects indicated CCRI35 contribute to the favorable alleles. QTLs were named according to their trait and the order on the chromosome. The region with three or more QTLs was regarded as QTL cluster.

### 4.6. Functional Annotation of Candidate Genes

We determined 99% confidence intervals of a stable QTL or QTL cluster as candidate regions. According to the annotation of *G. hirsutum* [17] and *G. darwinii* [24] reference genome, the genes in the candidate regions were obtained. Genes expressed in fibers or seeds were selected for further analysis based on the RNA-seq data of CCRI35 [10,23]. The expression profiles of the selected genes were analyzed using the R package mfuzz and visualized using the R-4.3.0 software. Gene Ontology annotations were performed on the Cotton Functional Genomics Database (CottonFGD) (https://cottonfgd.net/analyze/) [35].

## 5. Conclusions

In this study, 553 CSSLs with one or more segments of *G. tomentosum* were developed. Totals of 235 QTLs were identified for fiber- and seed-related traits. Of these, twenty-seven QTLs were detected in two or more environments, and the candidate genes for three of them were further identified. The results of this study provide a basis for exploring the molecular mechanism of fiber and seed development and marker-assisted genetic improvement in cotton.

## Figures and Tables

**Table 1:** QTL clusters identified in the CSSLs across multiple environments.

Cluster	QTLs
A01-cluster-1	*qPC_A01.1_*, *qFS_A01.1_*, *qFE_A01.1_*
A01-cluster-2	*qOA_A01.1_*, *qLP_A01.1_*, *qFL_A01.1_*, *qSI_A01.1_*, *qSA_A01.1_*, *qFM_A01.1_*, *qLA_A01.1_*
A02-cluster-1	*qSI_A02.1_*, *qOC_A02.1_*, *qSA_A02.1_*, *qLA_A02.1_*
A02-cluster-2	*qFL_A02.1_*, *qLA_A02.2_*, *qSA_A02.2_*, *qPC_A02.1_*, *qOC_A02.2_*, *qFU_A02.1_*
A03-cluster-1	*qFS_A03.1_*, *qFE_A03.1_*, *qOA_A03.1_*, *qLA_A03.1_*
A03-cluster-2	*qFL_A03.1_*, *qFS_A03.2_*, *qOC_A03.1_*, *qSA_A03.1_*, *qOA_A03.3_*, *qLA_A03.2_*
A05-cluster-1	*qFU_A05.1_*, *qPA_A05.1_*, *qSA_A05.2_*, *qLA_A05.1_*
A08-cluster-1	*qFS_A08.1_*, *qFM_A08.1_*, *qOC_A08.1_*, *qLP_A08.1_*
A09-cluster-1	*qPA_A09.1_*, *qOA_A09.1_*, *qLA_A09.1_*
A10-cluster-1	*qLP_A10.1_*, *qSI_A10.1_*, *qOC_A10.1_*, *qFE_A10.1_*, *qPA_A10.1_*
A10-cluster-2	*qPA_A10.2_*, *qLA_A10.2_*, *qPC_A10.1_*, *qOC_A10.2_*, *qLP_A10.2_*
A11-cluster-1	*qSI_A11.1_*, *qFS_A11.1_*, *qFE_A11.1_*, *qLP_A11.1_*
A11-cluster-2	*qFL_A11.1_*, *qFM_A11.1_*, *qPA_A11.1_*
A12-cluster-1	*qOC_A12.1_*, *qPA_A12.1_*, *qSA_A12.1_*
A12-cluster-2	*qFL_A12.3_*, *qFS_A12.2_*, *qLP_A12.1_*, *qSI_A12.1_*
D01-cluster-1	*qPC_D01.1_*, *qSI_D01.1_*, *qOA_D01.1_*, *qLA_D01.1_*, *qOC_D01.1_*
D01-cluster-2	*qOA_D01.2_*, *qPC_D01.2_*, *qLA_D01.2_*
D03-cluster-1	*qLP_D03.1_*, *qPC_D03.1_*, *qOC_D03.1_*, *qOA_D03.1_*, *qLA_D03.1_*, *qSI_D03.2_*, *qFM_D03.1_*, *qPA_D03.1_*, *qSA_D03.1_*
D05-cluster-1	*qLP_D05.1_*, *qFU_D05.1_*, *qOA_D05.1_*
D05-cluster-2	*qFL_D05.1_*, *qFS_D05.1_*, *qSI_D05.2_*
D08-cluster-1	*qFM_D08.2_*, *qPC_D08.1_*, *qOC_D08.1_*, *qOA_D08.1_*, *qLA_D08.1_*
D09-cluster-1	*qSI_D09.2_*, *qFS_D09.1_*, *qOC_D09.1_*, *qLP_D09.1_*
D10-cluster-1	*qLP_D10.1_*, *qFE_D10.1_*, *qFL_D10.1_*, *qFS_D10.1_*, *qSA_D10.2_*
D11-cluster-1	*qLP_D11.1_*, *qOA_D11.2_*, *qLA_D11.2_*, *qPC_D11.1_*, *qOC_D11.1_*, *qPA_D11.1_*, *qSA_D11.1_*
D12-cluster-1	*qLP_D12.1_*, *qFL_D12.1_*, *qSI_D12.1_*, *qPC_D12.1_*, *qPA_D12.1_*

## Data Availability

The raw datasets used for expression analysis of annotation genes were available at NCBI Sequence Read Archive database (https://submit.ncbi.nlm.nih.gov/about/sra/), with BioProject accession number PRJNA809429 and PRJNA1003140.

## Supplementary Materials

The following supporting information can be downloaded at: https://www.mdpi.com/article/10.3390/ijms25179639/s1.

## Abbreviations

QTL: quantitative trait loci; CSSLs, chromosome segment substitution lines; ILs, introgression lines; cM, centimorgan; Chr, chromosome; CQ, Chongqing; LP, lint percentage; SI, seed index; FL, fiber length; FS, fiber strength; FU, fiber uniformity; FM, fiber micronaire; FE, fiber elongation; PC, protein content; OC, oil content; PA, palmitic acid content; SA, stearic acid content; OA, oleic acid content; LA, linoleic acid content; GO, Gene Ontology; DPA, days post anthesis; ANOVA, analysis of variance; MQM, multiple-QTL model.

## References

[1] Khan N.U., Basal H., Hassan G. (2010). Cottonseed oil and yield assessment via economic heterosis and heritability in intraspecific cotton populations. Afr. J. Biotechnol..

[2] Lee J.H., Kim S.H. (2022). Synthesis and characterization of biopolyurethane crosslinked with castor oil-based hyperbranched polyols as polymeric solid-solid phase change materials. Sci. Rep..

[3] Shen X., Guo W., Zhu X., Yuan Y., Yu J., Kohel R., Zhang T. (2005). Molecular mapping of QTLs for fiber qualities in three diverse lines in Upland cotton using SSR markers. Mol. Breed..

[4] Zhang Z., Li J., Jamshed M., Shi Y., Liu A., Gong J., Wang S., Zhang J., Sun F., Jia F. (2020). Genome-wide quantitative trait loci reveal the genetic basis of cotton fibre quality and yield-related traits in a recombinant inbred line population. Plant Biotechnol. J..

[5] Wendel J.F., Grover C.E. (2015). Taxonomy and evolution of the cotton genus, *Gossypium*. Cotton.

[6] Liu X., Ma J., Li Q., Guo Z., Wang Y., Wang Q., Yao J., Zhang Y., Wang W., Teng Z. (2023). Detection of QTL controlling fiber-related traits in a recombinant inbred lines population from *G. hirsutum* race *punctatum* using RTM-GWAS procedure. Ind. Crops Prod..

[7] Wang B., Nie Y., Lin Z., Zhang X., Liu J., Bai J. (2012). Molecular diversity, genomic constitution, and QTL mapping of fiber quality by mapped SSRs in introgression lines derived from *Gossypium hirsutum* × *G. darwinii* Watt. Theor. Appl. Genet..

[8] Grover C.E., Zhu X., Grupp K.K., Jareczek J.J., Gallagher J.P., Szadkowski E., Seijo J.G., Wendel J.F. (2015). Molecular confirmation of species status for the allopolyploid cotton species, *Gossypium ekmanianum* Wittmack. Genet. Resour. Crop Evol..

[9] Chang X., Guo C., Pan Z., Wu Y., Shen C., Chao L., Shui G., You C., Xu J., Lin Z. (2023). QTL Mapping for fiber quality based on introgression lines population from *G. hirsutum* × *G. tomentosum*. Agriculture.

[10] Hao Y., Liu X., Wang Q., Wang S., Li Q., Wang Y., Guo Z., Wu T., Yang Q., Bai Y. (2024). Mapping QTL for fiber-and seed-related traits in *Gossypium tomentosum* CSSLs with G. hirsutum background. J. Integr. Agric..

[11] Wendel J.F., Percy R.G. (1990). Allozyme diversity and introgression in the Galapagos Islands endemic *Gossypium darwinii* and its relationship to continental *G. barbadense*. Biochem. Syst. Ecol..

[12] Liang Z. (1999). Genetic and Breeding Science of Cotton Hybridization.

[13] Jiang C.X., Chee P.W., Draye X., Morrell P.L., Paterson A.H. (2000). Multilocus interactions restrict gene introgression in interspecific populations of polyploid *Gossypium* (cotton). Evolution.

[14] Paterson A.H., Deverna J.W., Lanini B., Tanksley S.D. (1990). Fine Mapping of quantitative trait loci using selected overlapping recombinant chromosomes in an interspecies cross of tomato. Genetics.

[15] Young N.D., Tanksley S.D. (1989). RFLP analysis of the size of chromosomal segments retained around the Tm-2 locus of tomato during backcross breeding. Theor. Appl. Genet..

[16] Ali M.L., Sanchez P.L., Yu S.B., Lorieux M., Eizenga G.C. (2010). Chromosome segment substitution lines: A powerful tool for the introgression of valuable genes from *Oryza* wild species into cultivated rice (*O. sativa*). Rice.

[17] Hu Y., Chen J., Fang L., Zhang Z., Ma W., Niu Y., Ju L., Deng J., Zhao T., Lian J. (2019). *Gossypium barbadense* and *Gossypium hirsutum* genomes provide insights into the origin and evolution of allotetraploid cotton. Nat. Genet..

[18] Song J., Pei W., Wang N., Ma J., Xin Y., Yang S., Wang W., Chen Q., Zhang J., Yu J. (2022). Transcriptome analysis and identification of genes associated with oil accumulation in upland cotton. Physiol. Plant..

[19] Keerio A.A., Shen C., Nie Y., Ahmed M.M., Zhang X., Lin Z. (2018). QTL mapping for fiber quality and yield traits based on introgression lines derived from *Gossypium hirsutum* × *G. tomentosum*. Int. J. Mol. Sci..

[20] Lu Q., Li P., Yang R., Xiao X., Li Z., Wu Q., Gong J., Ge Q., Liu A., Du S. (2022). QTL mapping and candidate gene prediction for fiber yield and quality traits in a high-generation cotton chromosome substitution line with *Gossypium barbadense* segments. Mol. Genet. Genom..

[21] Wang B., Draye X., Zhuang Z., Zhang Z., Liu M., Lubbers E.L., Jones D., May O.L., Paterson A.H., Chee P.W. (2017). QTL analysis of cotton fiber length in advanced backcross populations derived from a cross between *Gossypium hirsutum* and *G. mustelinum*. Theor. Appl. Genet..

[22] Wang F., Zhang J., Chen Y., Zhang C., Gong J., Song Z., Zhou J., Wang J., Zhao C., Jiao M. (2020). Identification of candidate genes for key fibre-related QTLs and derivation of favourable alleles in *Gossypium hirsutum* recombinant inbred lines with *G. barbadense* introgressions. Plant Biotechnol. J..

[23] Yang P., Sun X., Liu X., Wang W., Hao Y., Chen L., Liu J., He H., Zhang T., Bao W. (2022). Identification of candidate genes for lint percentage and fiber quality through QTL mapping and transcriptome analysis in an allotetraploid interspecific cotton CSSLs population. Front. Plant Sci..

[24] Chen Z.J., Sreedasyam A., Ando A., Song Q., De Santiago LM D., Hulse-Kemp A.M., Ding M., Ye W., Kirkbride R.C., Jenkins J. (2020). Genomic diversifications of five *Gossypium* allopolyploid species and their impact on cotton improvement. Nat. Genet..

[25] Liu X., Yang L., Wang J., Wang Y., Guo Z., Li Q., Yang J., Wu Y., Chen L., Teng Z. (2022). Analyzing quantitative trait loci for fiber quality and yield-related traits from a recombinant inbred line population with *Gossypium hirsutum* race *palmeri* as one parent. Front. Plant Sci..

[26] Zhang M., Cao H., Xi J., Zeng J., Huang J., Li B., Song S., Zhao J., Pei Y. (2020). Auxin directly upregulates GhRAC13 expression to promote the onset of secondary cell wall deposition in cotton fibers. Front. Plant Sci..

[27] Qin Y., Sun M., Li W., Xu M., Shao L., Liu Y., Zhao G., Liu Z., Xu Z., You J. (2022). Single-cell RNA-seq reveals fate determination control of an individual fibre cell initiation in cotton (*Gossypium hirsutum*). Plant Biotechnol. J..

[28] Luzarowska U., Ruß A.-K., Joubès J., Batsale M., Szymański J., PThirumalaikumar V.P., Luzarowski M., Wu S., Zhu F., Endres N.J. (2023). Hello darkness, my old friend: 3-KETOACYL-COENZYME A SYNTHASE 4 is a branch point in the regulation of triacylglycerol synthesis in *Arabidopsis thaliana*. Plant Cell.

[29] Tan Z., Fang X., Tang S., Zhang J., Liu D., Teng Z., Li L., Ni H., Zheng F., Liu D. (2015). Genetic map and QTL controlling fiber quality traits in upland cotton (*Gossypium hirsutum* L.). Euphytica.

[30] Wang W., Tan Z., Xu Y., Zhu A., Li Y., Yao J., Tian R., Fang X., Liu X., Tian Y. (2017). Chromosome structural variation of two cultivated tetraploid cottons and their ancestral diploid species based on a new high-density genetic map. Sci. Rep..

[31] Voorrips R., Van D., Van Den Heuvel L., Ooijen J., Van J.W. (2006). JoinMap^®^ 4.0: Software for the Calculation of Genetic Linkage Maps in Experimental Populations.

[32] Tang H., Krishnakumar V., Zeng X., Xu Z., Taranto A., Lomas J.S., Zhang Y., Huang Y., Wang Y., Yim W.C. (2024). JCVI: A versatile toolkit for comparative genomics analysis. iMeta.

[33] Berloo R.V. (2008). GGT 2.0: Versatile software for visualization and analysis of genetic data. J. Hered..

[34] Van Ooijen J.W. (2009). MapQTL 6.0, Software for the Mapping of Quantitative Trait Loci in Experimental Populations of Dihaploid Species.

[35] Zhu T., Liang C., Meng Z., Sun G., Meng Z., Guo S., Zhang R. (2017). CottonFGD: An integrated functional genomics database for cotton. BMC Plant Biol..

