# Supplementary material for: QTL Mapping of Fiber- and Seed-Related Traits in Chromosome Segment Substitution Lines Derived from Gossypium hirsutum × Gossypium darwinii
Source: Int J Mol Sci. 2024 Sep 5;25(17):9639. doi: 10.3390/ijms25179639 (PMC11394887; doi:10.3390/ijms25179639)
Supplement: Supplementary file 1 [file ijms-25-09639-s001.zip › Supplemental file Figure S1-S3.pdf]

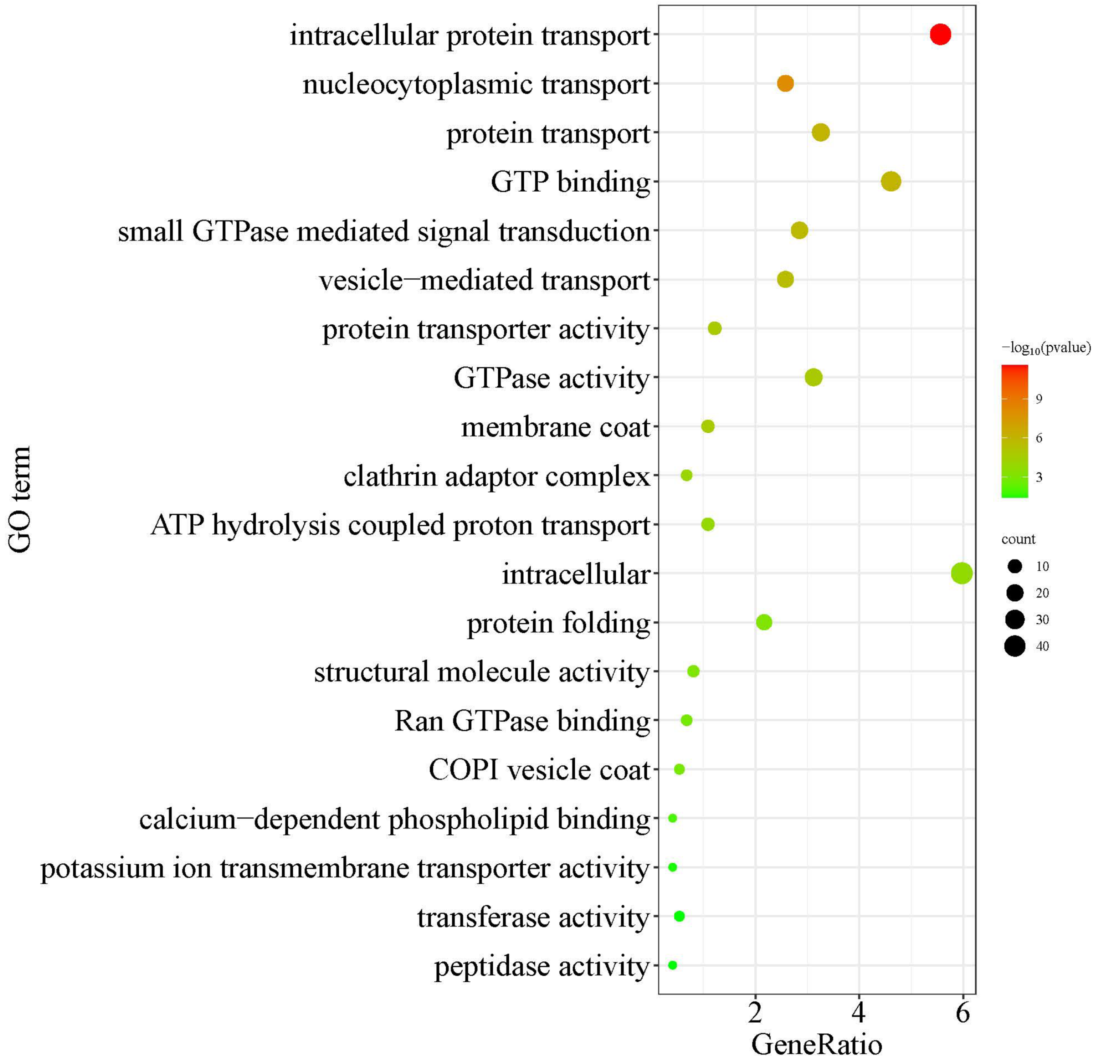

Figure S1. GO annotation of candidate genes associated with fiber elongation.

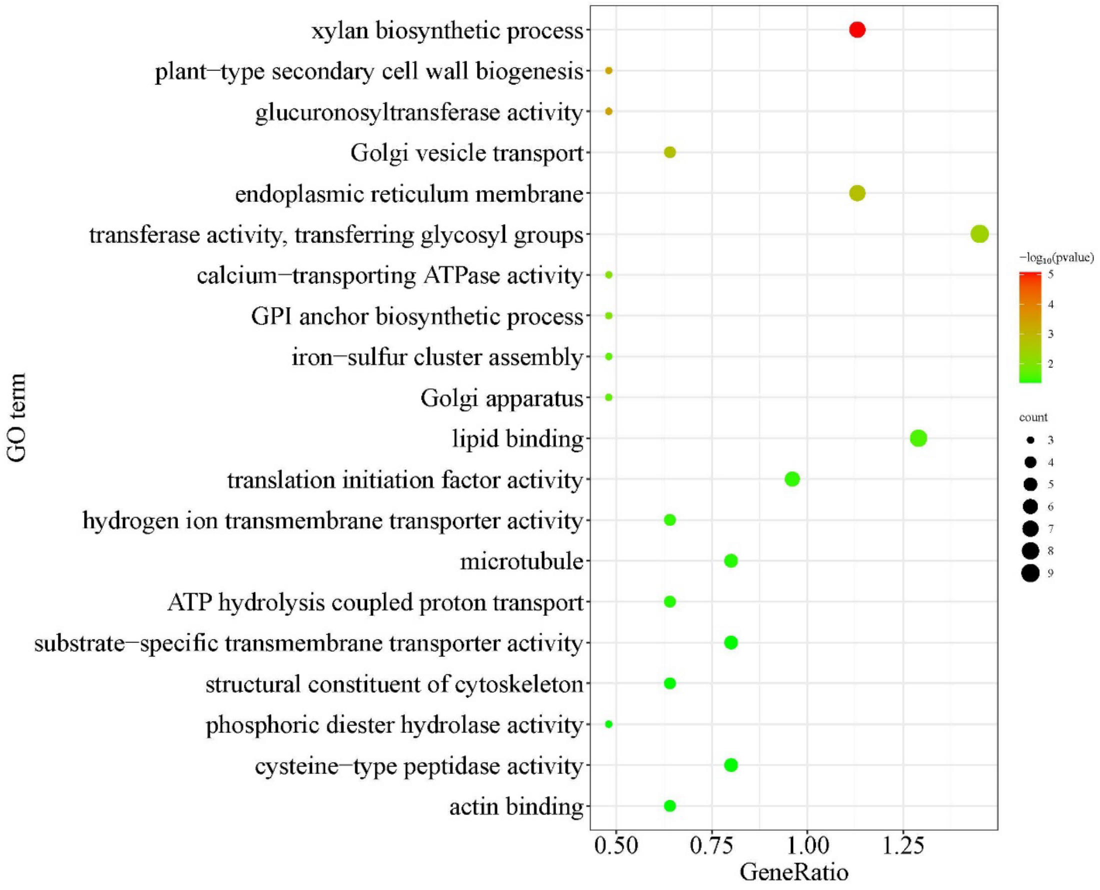

Figure S2. GO annotation of candidate genes associated with secondary wall deposition.

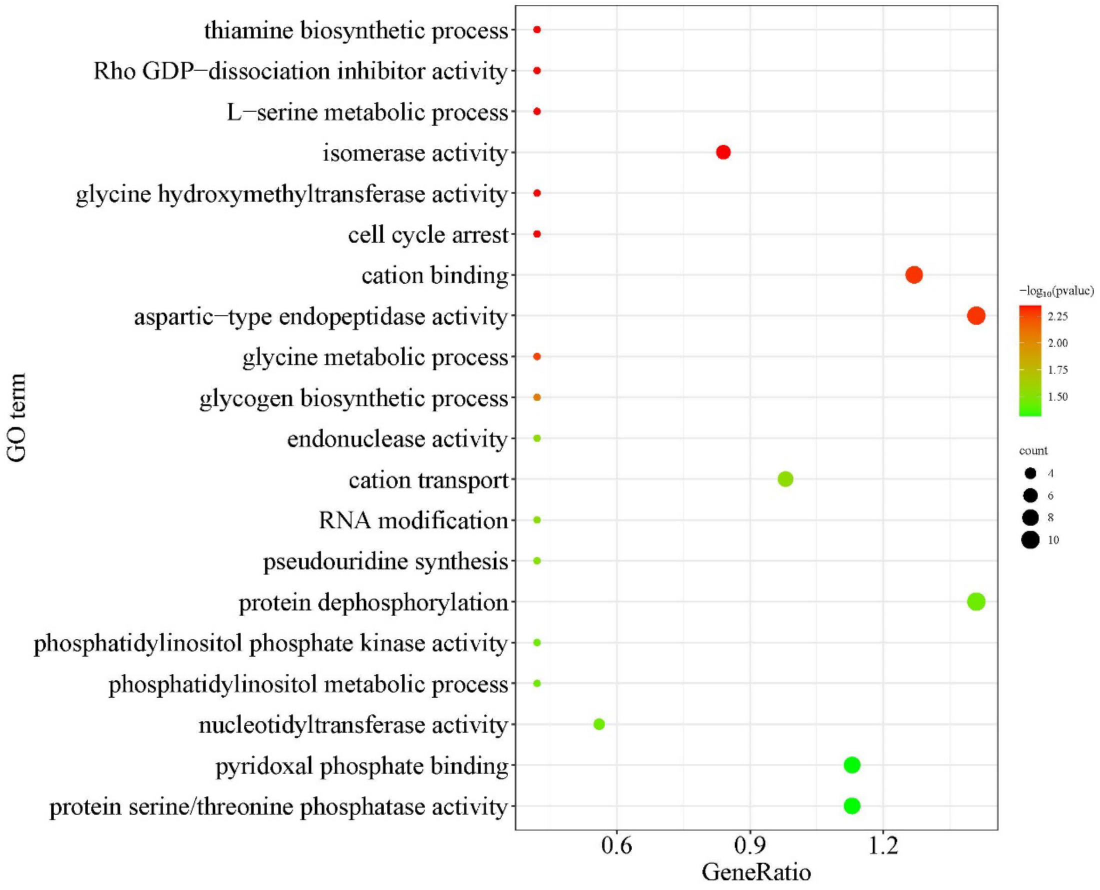

Figure S3. GO annotation of candidate genes associated with seed development.
